# Supplementary material for: Structurally distributed surface sites tune allosteric regulation
Source: eLife. 2021 Jun 16;10:e68346. doi: 10.7554/eLife.68346 (PMC8324303; doi:10.7554/eLife.68346)
Supplement: Supplementary file 1. — (a) Steady state kinetic parameters for select point mutants of the DL121 fusion. The parameter kcat is reported in units of s−1, Km is in units of µM. Error is calculated as standard error of the mean over three replicates. Related to Figure 4 of the main text. (b) Fisher Exact Test p-values for the null hypothesis that the sector and inactivating mutants are independent properties. Inactivating mutations are defined as those that yield relative growth rates at or below the growth rate for DL121-D27N. Over a range of sector definitions, the null hypothesis is rejected at a confidence level of 0.05 or better, shown in red. Sector definitions were taken from Reynolds et al., 2011 (Rivoire et al., 2016). (c) Fisher Exact Test p-values for the null hypothesis that conserved positions and inactivating mutants are independent. Calculations were made over a range of conservation definitions chosen to result in an equal number positions as the sector positions in Supplementary file 1b 23, 36, 40, and 49 positions respectively. In all cases, the null hypothesis is rejected at a confidence level of 0.05 or better (red), and inactivating mutations are enriched at conserved positions beyond expectation due to random chance. Conservation values are calculated as in Reynolds et al., 2011 (Rivoire et al., 2016), and reflect the Kullback-Leibler relative entropy of amino acid frequencies at each DHFR position. (d) Fisher Exact Test p-values for the null hypothesis that the sector and allosteric mutations are independent. We compared over four sector cutoffs (as defined in Rivoire et al., 2016) and at two cutoffs for allostery significance (a standard p-value of 0.05, and an adjusted p-value of 0.016). The multiple hypothesis testing adjusted p-value was obtained by Sequential Goodness of Fit (SGoF, Carvajal-Rodriguez and de Uña-Alvarez, 2011). The top table shows the association between sector positions and allostery enhancing mutations; the bottom table computes the associate bet [file elife-68346-supp1.docx]

| **Mutant** | ***K_cat_* lit** | **(+/-)** | **k_cat_ dark** | **(+/-)** | **K_m_**  **lit** | **(+/-)** | **K_m_ dark** | **(+/-)** | ***k_cat_*/K_m_**  **lit** | | **(+/-)** | ***k_cat_*/K_m_ dark** | **(+/-)** |
| --- | --- | --- | --- | --- | --- | --- | --- | --- | --- | --- | --- | --- | --- |
| Unmutated DL121 | 0.244 | 0.006 | 0.190 | 0.006 | 0.826 | 0.107 | 1.186 | 0.174 | 0.295 | 0.039 | | 0.160 | 0.024 |
| DL121 C450S | 0.221 | 0.007 | 0.197 | 0.006 | 0.822 | 0.141 | 0.833 | 0.128 | 0.269 | 0.047 | | 0.236 | 0.037 |
| DL121 A9N | 0.824 | 0.027 | 0.566 | 0.024 | 1.896 | 0.318 | 1.311 | 0.337 | 0.435 | 0.074 | | 0.432 | 0.113 |
| DL121 M16A | 0.241 | 0.005 | 0.134 | 0.006 | 0.972 | 0.118 | 1.036 | 0.258 | 0.247 | 0.030 | | 0.129 | 0.033 |
| DL121 R52K | 0.291 | 0.011 | 0.153 | 0.007 | 0.961 | 0.224 | 1.127 | 0.321 | 0.303 | 0.071 | | 0.135 | 0.039 |
| DL121 D87A | 0.334 | 0.011 | 0.173 | 0.005 | 0.724 | 0.138 | 0.627 | 0.115 | 0.462 | 0.089 | | 0.276 | 0.051 |
| DL121 E120P | 0.491 | 0.015 | 0.278 | 0.009 | 1.385 | 0.205 | 0.833 | 0.157 | 0.354 | 0.054 | | 0.333 | 0.064 |
| DL121 D122W | 0.124 | 0.003 | 0.106 | 0.003 | 1.238 | 0.174 | 1.719 | 0.269 | 0.100 | 0.014 | | 0.061 | 0.010 |
| DL121 H124Q | 0.291 | 0.010 | 0.144 | 0.005 | 1.118 | 0.193 | 1.003 | 0.167 | 0.260 | 0.046 | | 0.144 | 0.024 |
| DL121 D127W | 0.288 | 0.009 | 0.171 | 0.005 | 0.388 | 0.088 | 0.857 | 0.136 | 0.741 | 0.170 | | 0.199 | 0.032 |
| DL121 M16A,H124Q | 0.202 | 0.003 | 0.063 | 0.003 | 0.683 | 0.136 | 0.153 | 0.043 | 0.295 | 0.059 | | 0.409 | 0.116 |
| DL121- M16A,D87A,H124Q | 0.171 | 0.003 | 0.044 | 0.002 | 0.828 | 0.193 | 0.519 | 0.135 | 0.207 | 0.048 | | 0.084 | 0.022 |

**Supplementary File 1a**

| **Cutoff for sector definition:** | 0.005 | 0.008 | 0.01 | 0.015 |
| --- | --- | --- | --- | --- |
| Inactivating mutations in sector | 223 | 332 | 373 | 448 |
| Expected by chance | 177 | 280 | 311 | 381 |
| p-value: | 5.73 x 10^-7^ | 2.34 x 10^-6^ | 7.88 x 10^-8^ | 3.19 x 10^-8^ |
|  |  |  |  |  |

**Supplementary File 1b**

| **Cutoff for Conserved Positions:** | 1.89 | 1.54 | 1.49 | 1.38 |
| --- | --- | --- | --- | --- |
| Conserved inactivating mutations | 249 | 377 | 413 | 494 |
| Expected by chance | 175 | 276 | 309 | 374 |
| p-value: | 7.26 x 10^-16^ | 3.78 x 10^-20^ | 8.70 x 10^-20^ | 6.60 x 10^-23^ |
|  |  |  |  |  |

**Supplementary File 1c**

|  | **Sector Cutoff:** | 0.005 | 0.008 | 0.01 | 0.015 |
| --- | --- | --- | --- | --- | --- |
| **Cutoff for allostery enhancing:** | Allosteric positions in sector: | 4 | 5 | 5 | 7 |
|  | Expected | 13 | 22 | 25 | 31 |
| p < 0.05 | p-value: | 0.008 | 3.43 x 10^-5^ | 5.82 x 10^-6^ | 3.52 x 10^-7^ |
| p < 0.016 | # in sector: | 0 | 0 | 0 | 1 |
|  | Expected | 6 | 11 | 12 | 15 |
|  | p-value: | 0.013 | 4.23 x 10^-4^ | 1.68 x 10^-4^ | 4.64 x 10^-5^ |
|  |  |  |  |  |  |
|  |  |  |  |  |  |
|  | **Sector Cutoff:** | 0.005 | 0.008 | 0.01 | 0.015 |
| **Cutoff for allostery disrupting:** | Allosteric positions in sector: | 2 | 15 | 15 | 15 |
|  | Expected | 5 | 8 | 9 | 11 |
| p < 0.05 | p-value: | 0.257 | 0.013 | 0.038 | 0.25 |
| p < 0.016 | # in sector: | 1 | 7 | 7 | 7 |
|  | Expected | 1 | 2 | 3 | 3 |
|  | p-value: | 0.967 | 0.004 | 0.010 | 0.049 |

**Supplementary File 1d**

|  | **Allosteric Surface Positions** | |
| --- | --- | --- |
| **Cutoff for allostery enhancing:** | Observed | 88 |
|  | Expected | 73 |
| p < 0.05 | p-value: | 0.004 |
| p < 0.016 | Observed | 44 |
|  | Expected | 35 |
|  | p-value: | 0.013 |
|  |  |  |
|  |  |  |
|  | **Allosteric Surface Positions** | |
| **Cutoff for allostery disrupting:** | Observed | 33 |
|  | Expected | 27 |
| p < 0.05 | p-value: | 0.061 |
| p < 0.016 | Observed | 12 |
|  | Expected | 8 |
|  | p-value: | 0.048 |

**Supplementary File 1e**

|  | **Sector Cutoff:** | 0.005 | 0.008 | 0.01 | 0.015 |
| --- | --- | --- | --- | --- | --- |
| **Cutoff for allostery enhancing:** | Allosteric surface positions within or connected to sector: | 2 | 13 | 15 | 28 |
|  | Expected | 18 | 27 | 28 | 33 |
| p < 0.05 | p-value: | 4.88 x 10^-5^ | 0.002 | 0.006 | 0.360 |
| p < 0.016 | # connected: | 10 | 5 | 7 | 15 |
|  | Expected | 1 | 13 | 13 | 16 |
|  | p-value: | 0.009 | 0.019 | 0.074 | 0.987 |
|  |  |  |  |  |  |
|  |  |  |  |  |  |
|  | **Sector Cutoff:** | 0.005 | 0.008 | 0.01 | 0.015 |
| **Cutoff for allostery disrupting:** | Allosteric surface positions within or connected to sector: | 19 | 22 | 22 | 24 |
|  | Expected | 6 | 10 | 10 | 12 |
| p < 0.05 | p-value: | 1.56 x 10^-7^ | 1.52 x 10^-5^. | 2.72 x 10^-5^. | 6.60 x 10^-5^ |
| p < 0.016 | # connected: | 8 | 9 | 9 | 10 |
|  | Expected | 2 | 3 | 3 | 4 |
|  | p-value: | 1.38 x 10^-5^ | 2.30 x 10^-4^ | 3.16 x 10^-4^ | 2.50 x 10^-4^ |

**Supplementary File 1f**

|  | **Sector Cutoff:** | 0.005 | 0.008 | 0.01 | 0.015 |
| --- | --- | --- | --- | --- | --- |
| **Cutoff for allostery enhancing:** | Allosteric surface positions connected to sector: | 2 | 13 | 15 | 28 |
|  | Expected | 13 | 18 | 17 | 19 |
| p < 0.05 | p-value: | 0.001 | 0.269 | 0.697 | 0.033 |
| p < 0.016 | # connected: | 1 | 5 | 7 | 15 |
|  | Expected | 6 | 8 | 8 | 9 |
|  | p-value: | 0.041 | 0.275 | 0.837 | 0.049 |
|  |  |  |  |  |  |
|  |  |  |  |  |  |
|  | **Sector Cutoff:** | 0.005 | 0.008 | 0.01 | 0.015 |
| **Cutoff for allostery disrupting:** | Allosteric surface positions connected to sector: | 18 | 7 | 7 | 9 |
|  | Expected | 5 | 6 | 6 | 7 |
| p < 0.05 | p-value: | 3.83 x 10^-10^ | 0.971 | 0.883 | 0.379 |
| p < 0.016 | # connected: | 8 | 2 | 2 | 3 |
|  | Expected | 1 | 2 | 2 | 2 |
|  | p-value: | 8.02 x 10^-8^ | 0.731 | 0.772 | 0.777 |

**Supplementary File 1g**
