# Supplementary material for: Structurally distributed surface sites tune allosteric regulation
Source: eLife. 2021 Jun 16;10:e68346. doi: 10.7554/eLife.68346 (PMC8324303; doi:10.7554/eLife.68346)
Supplement: Transparent reporting form [file elife-68346-transrepform.docx]

***eLife’s* transparent reporting form**

We encourage authors to provide detailed information *within their submission* to facilitate the interpretation and replication of experiments. Authors can upload supporting documentation to indicate the use of appropriate reporting guidelines for health-related research (see [EQUATOR Network](http://www.equator-network.org/%20)), life science research (see the [BioSharing Information Resource](https://biosharing.org/)), or the [ARRIVE guidelines](http://www.plosbiology.org/article/info:doi/10.1371/journal.pbio.1000412) for reporting work involving animal research. Where applicable, authors should refer to any relevant reporting standards documents in this form.

If you have any questions, please consult our Journal Policies and/or contact us: [editorial@elifesciences.org](mailto:editorial@elifesciences.org).

**Sample-size estimation**

- You should state whether an appropriate sample size was computed when the study was being designed
- You should state the statistical method of sample size computation and any required assumptions
- If no explicit power analysis was used, you should describe how you decided what sample (replicate) size (number) to use

N/A; samples were time series of *E. coli* populations representing 10^8^-10^9^ cells and biochemical protein kinetic measurements.

Please outline where this information can be found within the submission (e.g., sections or figure legends), or explain why this information doesn’t apply to your submission:

**Replicates**

- You should report how often each experiment was performed
- You should include a definition of biological versus technical replication
- The data obtained should be provided and sufficient information should be provided to indicate the number of independent biological and/or technical replicates
- If you encountered any outliers, you should describe how these were handled
- Criteria for exclusion/inclusion of data should be clearly stated
- High-throughput sequence data should be uploaded before submission, with a private link for reviewers provided (these are available from both GEO and ArrayExpress)

Please outline where this information can be found within the submission (e.g., sections or figure legends), or explain why this information doesn’t apply to your submission:

For sequenced bacterial growth rate experiments we performed three (figure 3-5) or four (figure 2) biological replicates, where a replicate reflects growth of a mixed population in a single culture tube. Detailed information is in the Methods: “Growth Rate Measurements in the Turbidostat for DHFR DL121 Mutant Library”, “Growth Rate Measurements in the Turbidostat for DHFR Control Library”, “Plate reader assay for E. coli growth” - each had an n of 3, 4, and 6, respectively. No replicate was excluded from the data.

In sequencing experiments, probabilistic Illumina sequencer error correction was performed in the supplied Hamming_analysis.ipynb script as well as described in the “Read Joining, Filtering, and Counting” section in Methods.

Sequence data is available at: <https://www.ncbi.nlm.nih.gov/bioproject/PRJNA706683>

For steady state Michaelis Menten kinetics three replicates were performed for every substrate concentration (minimum of 8), the full curves are shown in figures 1C, S8, and S12, and kinetics parameters (with standard error of the mean) are given in Table S1.

**Statistical reporting**

- Statistical analysis methods should be described and justified
- Raw data should be presented in figures whenever informative to do so (typically when N per group is less than 10)
- For each experiment, you should identify the statistical tests used, exact values of N, definitions of center, methods of multiple test correction, and dispersion and precision measures (e.g., mean, median, SD, SEM, confidence intervals; and, for the major substantive results, a measure of effect size (e.g., Pearson's r, Cohen's d)
- Report exact p-values wherever possible alongside the summary statistics and 95% confidence intervals. These should be reported for all key questions and not only when the p-value is less than 0.05.

Please outline where this information can be found within the submission (e.g., sections or figure legends), or explain why this information doesn’t apply to your submission:

Figure 4A describes both the statistical test (t-test) and multiple hypothesis testing p-value adjustment used to assess statistically significant changes in allostery. Individual data points (raw data) for significant mutants are shown in 4B. Exact p-values are reported in tables S2-S7. The analysis method used for each experiment is reported in the methods section.

For kinetics experiments the full curves are shown in figures 1C, S8, and S1 with error bars representing the standard deviation. The fit to a Michaelis Menten model using non-linear regression is detailed in “Steady state Michaelis Menten Measurements”. The resulting kinetic parameters are detailed in Table S1 including the standard error of the mean.

(For large datasets, or papers with a very large number of statistical tests, you may upload a single table file with tests, Ns, etc., with reference to sections in the manuscript.)

**Group allocation**

- Indicate how samples were allocated into experimental groups (in the case of clinical studies, please specify allocation to treatment method); if randomization was used, please also state if restricted randomization was applied
- Indicate if masking was used during group allocation, data collection and/or data analysis

Please outline where this information can be found within the submission (e.g., sections or figure legends), or explain why this information doesn’t apply to your submission:

N/A; samples were time series of *E. coli* populations and biochemical protein kinetic measurements. More completely addressed in methods section.

**Additional data files (“source data”)**

- We encourage you to upload relevant additional data files, such as numerical data that are represented as a graph in a figure, or as a summary table
- Where provided, these should be in the most useful format, and they can be uploaded as “Source data” files linked to a main figure or table
- Include model definition files including the full list of parameters used
- Include code used for data analysis (e.g., R, MatLab)
- Avoid stating that data files are “available upon request”

Please indicate the figures or tables for which source data files have been provided:

[Sequence data](https://trace.ncbi.nlm.nih.gov/Traces/sra/?study=SRP309306) and [code used](https://github.com/reynoldsk/allostery-in-dhfr) for data analysis is linked to in “Data and Materials Availability section”. The code contains the scripts used to convert the raw reads to mutant counts (also included as text files), calculate allostery, and perform statistical analysis, as well as generate the raw figures as PDF files for figures 3-6, S1-4, S6, S7, and data for supplemental tables 2-6.
